# Assessing the health impacts of peatland fires: a case study for Central Kalimantan, Indonesia

**DOI:** 10.1007/s11356-019-06264-x

**Published:** 2019-08-30

**Authors:** Saritha Kittie Uda, Lars Hein, Dwi Atmoko

**Affiliations:** 1 Environmental Systems Analysis Group, Wageningen University & Research, Droevendaalsesteeg 3, 6708 PB Wageningen, The Netherlands; 2 Biology Study Program, University of Palangka Raya, Jl. Yos Sudarso, Palangka Raya, Central Kalimantan 73111A Indonesia; 3 Indonesian Agency for Meteorological Climatological and Geophysics, Badan Meteorologi Klimatologi dan Geofisika (BMKG), Jl. Angkasa I No.2 Kemayoran, Jakarta Pusat, DKI, Jakarta, 10720 Indonesia

**Keywords:** Tropical peatland fires, Smoke dispersion, PM_2.5_ concentration, Indonesia, Human health impacts

## Abstract

The conversion of Indonesian tropical peatlands has been associated with the recurring problems of peatland fires and smoke affecting humans and the environment. Yet, the local government and public in the affected areas have paid little attention to the impacts and costs of the poor air quality on human health. This study aims to analyse the long-term health impacts of the peat smoke exposure to the local populations. We applied the Hybrid Single-Particle Lagrangian Integrated Trajectory (HYSPLIT) model to determine the smoke dispersion and the associated PM_2.5_ concentrations of the resulted plumes from the fire hotspots in the deep and shallow peatlands in Central Kalimantan, Indonesia, that occurred during a 5-year period (2011–2015). We subsequently quantified the long-term health impacts of PM_2.5_ on the local people down to the village level based on the human health risk assessment approach. Our study shows that the average increase in the annual mean PM_2.5_ concentration due to peatland fires in Central Kalimantan was 26 μg/m^3^ which is more than twice the recommended value of the World Health Organisation Air Quality Guidelines. This increase in PM_2.5_ leads to increased occurrence of a range of air pollution–related diseases and premature mortality. The number of premature mortality cases can be estimated at 648 cases per year (26 mortality cases per 100,000 population) among others due to chronic respiratory, cardiovascular and lung cancer. Our results shed further light on the long-term health impacts of peatland fires in Indonesia and the importance of sustainable peatland management.

## Introduction

Smoke from peatland fires is a significant air pollution source associated with harmful impacts on human health and the environment. In Indonesia, peatland fires are mostly anthropogenic that may be started by farmers as part of small-scale land clearing activities, and by private companies to prepare for plantation establishment (Miettinen et al. 2017; Uda et al. 2017; Atwood et al. 2016; Turetsky et al. 2015; Marlier et al. 2015). In particular in the dry season, peat fires are difficult to control and may spread well beyond the area of ignition. Because of incomplete burning, peatland fires strongly contribute to emission of smoke haze pollutants, which contain a mixture of (fine and coarse) particulate matters or roots and various toxic and non-toxic gases (Stockwell et al. 2016). During the peatland fire episodes, in particular during the dry seasons in El Niño years, smoke covers major parts of Indonesia and even neighbouring countries (Tacconi 2016; Crippa et al. 2016). This results in negative impacts on people’s health and imposes substantial costs to society. Reported impacts include general negative health effects; disruption on transportation (flights, road trips) and tourism business; reduced enjoyment and quality of life; increased production of ozone, acid rain, and greenhouse gases; biodiversity loss; and reduced photosynthesis in plants because of the blocked solar radiation (Benjamin et al. 2017; World Bank 2016; Koplitz et al. 2016; Hirano et al. 2012).

To further specify the impacts of peat fires, in the El Niño year of 2015 approximately 4.6 million hectares were burned, with 37% located on peatland areas, and half of the total burned area was in Kalimantan (Lohberger et al. 2018). During the period August–November 2015, many parts of Indonesia, particularly in Kalimantan and Sumatra, were reported to be heavily blanked in thick smoke (Stockwell et al. 2016). The average daily CO_2_ emissions over the Maritime southeast Asia region (including Indonesia, Malaysia, Singapore) during the 2015 Indonesia forest and peatland fires (biomass burning) reached 11.3 TgCO_2_. This figure surpassed the daily release of CO_2_ from fossil fuel burning in the European Union (8.9 Tg CO_2_ per day) (Huijnen et al. 2016). The fires also led to very high atmospheric particulate matter (PM) concentrations. For instance, in Central Kalimantan province, the Pollutant Standards Index (PSI) of fine particulate matter (PM_2.5_) had been reported to exceed 1500 (PM_2.5_ > 1250 μg/m^3^), considerably above short-term exposure levels considered hazardous for human health (PSI > 300, PM_2.5_ > 250 μg/m^3^) (Atwood et al. 2016). The health effects of the inhalable PM both in short-term and long-term are well documented which include respiratory and cardiovascular morbidity (e.g. aggravation of asthma, respiratory symptoms and an increase in hospital admissions) and mortality from cardiovascular and respiratory diseases and from lung cancer (WHO 2013).

Indonesia lacks real-time and regional air quality data due to the absence of an integrated air quality monitoring network. The air quality monitoring stations are sparse which results in insufficient data about high-risk air pollution exposures, thereby limiting the assessment of the severity of the fire-related air pollution episodes. Although the air quality conditions and the associated public health outcomes (e.g. mortality) of Indonesian forest and peatland fires have been estimated (e.g. Koplitz et al. 2016; Crippa et al. 2016; Ruchi and Rajasekhar 2017), there is still a lack of information about the potential short- and long-term related diseases at the local scale in this country (Carmenta et al. 2017). Consequently, local governments and communities in the affected areas have paid little attention to the impacts and costs of the poor air quality on the human health and environment that are caused by the mentioned annual peatland fires (Sumarga 2017; Uda et al. 2018).

This study aims to estimate the human health outcomes of the long-term exposure to peat smoke in the province of Central Kalimantan. The results can inform policymakers and stakeholders (including peatland users) on the urgency of tackling (recurrence) peatland fires and also help to increase public awareness on the importance of healthy air quality. We considered peatland fire evidences from Central Kalimantan during a 5-year period (2011, 2012, 2013, 2014 and 2015) and conducted a literature review and spatial analysis to analyse the smoke dispersion in order to estimate the annual PM_2.5_ concentrations of the peatland fires from the deep and shallow peatland areas. We assume that the conditions during this 5-year period are representative of the long-term conditions. Subsequently, we assess long-term effects of PM_2.5_ exposure to local people’s health based on the average concentration in these 5 years, assuming that this period, which includes one El Nino year, is representative for people’s long-term exposure.

## Material and methods

### Study area

**Fig. 1:**
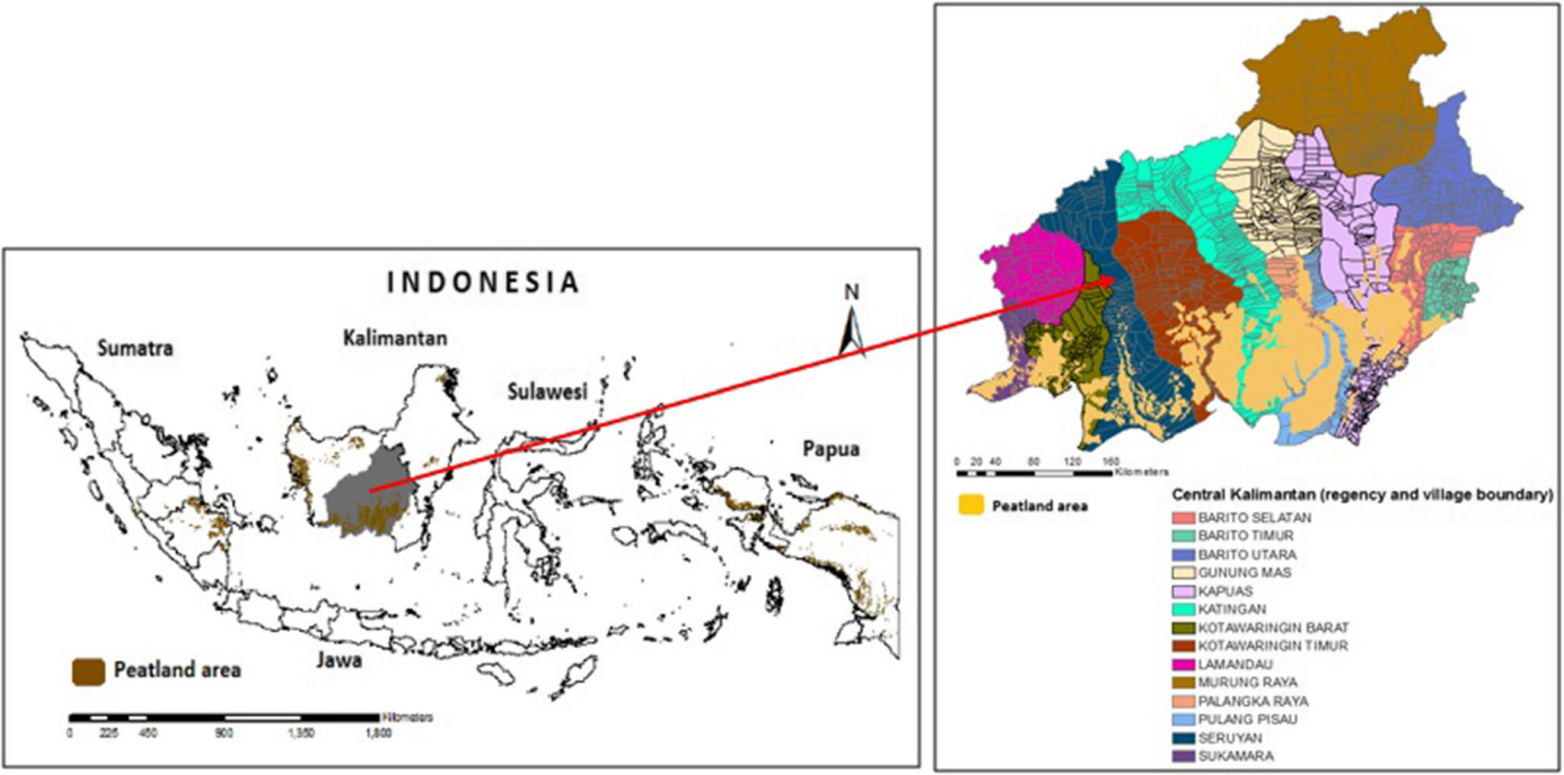
Indonesian peatland distribution map (Ritung et al. 2011); Central Kalimantan province as the study area is in grey, covering 14 regencies

Indonesia has about 14.9 million hectares of tropical peatlands (about 8% of its total land area) that are mainly distributed across the regions of Sumatra, Kalimantan and Papua. This study specifically focuses on Central Kalimantan Province, Indonesia (see Fig. 1), which comprises about 56% of the total peatland area of the Kalimantan island and about 18% of the total Indonesia peatlands (Ritung et al. 2011). Central Kalimantan is the third largest province in Indonesia, located between latitudes 0° 45′ North and 3° 30′ South, and longitudes 110° 45′–115° 51′ East, with a total area of 153,564 km^2^. It has about 2.7 million hectares of peatland areas (about 18% of the total Central Kalimantan Province area), of which 59% is deep peatlands (over 3-m deep). Central Kalimantan Province covers 14 regencies (about 1569 villages), with a population of approximately 2.5 million people (BPS Central Kalimantan 2016). Central Kalimantan has approximately 13 million hectares of forest areas (INCAS 2016). However, over the past 20 years, the forest and peatland areas in this province have been converted extensively due to land use changes and annual fires from land clearing which have been contributing significantly to the total greenhouse emissions in Indonesia (Miettinen et al. 2016; Sumarga 2017).

### Analysis of smoke dispersion and associated PM_2.5_ concentration of peat smoke

To estimate the long-term health effects of peat smoke, we first calculated the increase in annual concentration of PM_2.5_ resulting from peat fire hotspots in Central Kalimantan peatland areas during 2011–2015. We assumed that the 5-year period of 2011–2015 (that includes one El Niño year) is representative for the long-term concentration and exposure of peat smoke on the people living in the areas. We randomly selected 200 fire hotspots each year (100 in deep and 100 in shallow peat) that occurred in the peatlands located in Central Kalimantan, and subsequently we used the processed data as input for the smoke dispersion model that was used to analyse the associated PM_2.5_ concentration. Fire hotspots are identified with the MODIS Aqua/Terra sensor, and smoke plumes were aggregated to obtain a map depicting the distribution of smoke over Central Kalimantan. We scaled up the smoke concentration by multiplying the found, averaged, smoke concentration caused by a peat fire by the number of hotspots occurring in shallow respectively deep peat in a given year, and further calibrated the model to the Palangka Raya air quality monitoring site (there is only one air quality measurement station in Central Kalimantan, which provides daily estimates of PM_2.5_ concentrations). We combined this with information on population density and thereby local exposures to PM_2.5_, and subsequently estimated the long-term health effects for the local populations on village-based analysis.

In order to generate a Central Kalimantan peatland map, we overlaid the Indonesia Peatland Map Scale 1:250,000, produced by Balai Besar Sumber Daya Lahan Pertanian (BBSDLP), the Ministry of Agriculture Republic Indonesia (Ritung et al. 2011), with the Central Kalimantan Land Cover Map, produced by the Ministry of Forestry Republic Indonesia (MoFRI 2014). Next, we overlaid the aforementioned result with the burned area and the hotspot datasets from 2011 to 2015 (containing information about latitude and longitude coordinates, date and time, confidence values; obtained from the MODIS Aqua/Terra satellites) that was obtained from the Sipongi output programs by Ministry of Environment and Forestry Republic Indonesia (MoEFRI 2015) and the Lembaga Penerbangan dan Antariksa Nasional/LAPAN (The Indonesian National Institute of Aeronautics and Space 2015). The hotspots indicate the temperatures in a specific sensor element above a certain threshold that are defined as active fire events (burning material on the surface). Here, the MODIS sensor defines a hotspot as a detected temperature above 47 °C located within a spatial resolution of about 1 km^2^ (Giglio 2015; MoEFRI 2015). We only constrained the data with a confidence degree of hotspot equal to 80–100% corresponding to the high likelihood of real fires (in line with Giglio 2015; The Indonesian National Institute of Aeronautics and Space 2015). Based on this result, we then analysed the distribution of the hotspots in the deep and shallow peatland areas in Central Kalimantan. In total, there are about 3155, 3604, 1246, 7454 and 21,408 hotspots in peatland areas that were recorded during the year of 2011, 2012, 2013, 2014 and 2015 respectively. For the purpose of this study, it is impossible to analyse all of these hotspots, so we analysed 200 randomly selected hotspots each year and we analysed and averaged them to define an ‘average’ smoke plumes of a peat fire hotspot in a given year. Of the selected 200 hotspots, 100 hotspots were selected to occur in shallow peatlands and 100 hotspots in deep peatlands. We followed code of peat depth by Ritung et al. (2011) to distinguish these hotspots in the peatlands, i.e. codes of D1 and D2 are for the shallow peatlands (50–200-cm peat depth) and codes of D3 and D4 are for the deep peatlands (> 200-cm peat depth). The monthly hotspot data were extracted, and those with confidence degree ≥ 80 were then selected and overlaid on the peat map.

Next, we applied the Hybrid Single-Particle Lagrangian Integrated Trajectory model (HYSPLIT version 4.9) to determine the atmospheric dispersions and plume trajectory as well as the PM_2.5_ concentration produced by each selected hotspot. With the HYSPLIT model, we estimated the spatial and temporal evolution of PM_2.5_ from a prescribed burn using the location and the burned area as inputs (Stein et al. 2015). The Global Data Assimilation System (GDAS) with a horizontal resolution of 0.5° was used as the meteorological and emission data input. GDAS is daily archive files that contain global 3-dimensional gridded meteorological model output. The files contain 3-hourly data, at a half-degree latitude by half-degree longitude with resolution 720 × 361 grid points, on 55 hybrid sigma-pressure surfaces. Here, the HYSPLIT model does not take into account the effect of the following: chemical reactions; dense gases; byproducts from fires, explosions, or chemical reactions; complex terrain—other than what is resolved by the meteorological model’s terrain (see https://ready.arl.noaa.gov/hypub/limitations.html). We also applied several assumptions in the HYSPLIT modelling for the runtime and deposition parameters. This included 24 hours for the total duration of transported pollutant material downwind (mostly peatland fires in Central Kalimantan were more than 24 hours), 24 hours for the pollutant averaging period (output interval of concentration released), 100 m AGL (above-ground-level) for the top averaged plume’s layer (100 m AGL is the minimum height to adequately represent the plume and indicate the concentration), and deposition parameters for the dry deposition rate (0.001 m/s) and for the wet deposition rate (8.0E-05 litter/s). The output of the HYSPLIT model shows the dispersion within the direction of the plumes, with the range concentrations of PM_2.5_ (including the maximum and minimum concentrations).

We then aggregated all the plumes of “observed hotspots” resulting from the HYSPLIT model and adjusted the resulting PM_2.5_ concentrations by calibrating them with the annual average concentration of PM_2.5_ in Palangka Raya City for the total amount of hotspots in peatland areas during 2011–2015. We used these average concentrations of PM_2.5_ to estimate the annual concentration of PM_2.5_ of all observed hotspots in a given year. The resulting PM_2.5_ concentrations were aggregated (spatially) in order to produce a map of annual mean concentration of PM_2.5_ for a 5-year period (2011–2015). The ground-based observation data used for the calibration was taken from the Air Quality Monitoring System/AQMS (or Indeks Standar Pencemaran Udara/ISPU), published by the Environmental Agency (Badan Lingkungan Hidup Daerah/BLHD) of Palangka Raya City, Central Kalimantan, and Badan Meteorologi Klimatologi dan Geofisika/BMKG (the Indonesian Agency for Meteorological Climatological and Geophysics). By using the Central Kalimantan administration map (published by Central Kalimantan Statistical Bureau), we spatially quantified the annual average concentration of PM_2.5_ in every village based on the village boundaries. We subsequently used this output to assess the exposure of people to PM_2.5_ on a village-based analysis as described in the next step. All spatial analyses were implemented using ArcGIS 10.5 at a spatial resolution of 1-km^2^ grid cell and with the output coordinate system of WGS 1984 UTM Zone 49S. All of the HYSPLIT models were done using NOAA ARL (Air Resources Laboratory NOAA) software (Stein et al. 2015).

### Analysis of the long-term human health impacts of the PM_2.5_ exposure

In order to analyse the mortality impacts of PM_2.5_ exposure resulting from the peat smoke, we quantified the health impacts (number of premature mortality cases including total mortality and mortality due to different diseases) in the receptors (inhabitants) in each village. We applied a human health risk assessment based on Ostro (2004), Burnett et al. (2014), Crippa et al. (2016) and Koplitz et al. (2016). We calculated the relative risk (RR) and the attributable fraction (AF or impact fraction, IF) of premature mortality for three types of the health case categories, i.e. cardiovascular, lung cancer and chronic respiratory diseases due to long-term exposure to PM_2.5_ (Crippa et al. 2016). We applied the log-linear exposure formula for the relative risk function as RR *=* [(*X* + 1)/(*X*_*o*_ + 1)]^*β*^ for *X ≥X*_*o*_, where *X* refers to the average of the annual mean concentration of PM_2.5_ (in μg/m^3^), during the period of observation. *X*_*o*_ is the lowest observed concentration from the average of annual mean of PM_2.5_ concentration (μg/m^3^, as the lowest effect level) and *β* is the excess mortality per-unit increase in PM_2.5_ with suggested *β* coefficients of 0.1551, 0.23218, 0.003794 and 0.001829 for measuring cardiovascular case, lung cancer case, chronic respiratory case and premature mortality, respectively (Ostro 2004). For the purpose of this study, we renormalize the suggested *β* coefficient for all-cause of mortality (0.0008) and chronic respiratory case due to PM_10_ exposure (0.00166) by multiplying the coefficient with the Indonesia conversion factor 48/21 (PM_10_/PM_2.5_ ratio) (WHO 2014). Next, we calculated the attributable fraction by using AF function as AF_d_*=* RR_d_ (*X*) − RR_d_ (*X*_*o*_) where RR_d_ is relative risk of disease (Crippa et al. 2016). The total number of mortality cases due to long-term exposure of PM_2.5_ from peatland fires and smoke in the study area were calculated by multiplying the attributable fraction (AF) with the baseline mortality risk of the related health case and the number of population in the study area (Ostro 2004; Koplitz et al. 2016; Crippa et al. 2016).

It is noted that in this study, the baseline mortality rate is based on the overall death rate (CDR) for Central Kalimantan in 2015 which was 5.8 per 1000 population of all ages (BPS Central Kalimantan 2017), reflecting a still growing population. We used village-based data for year 2015 provided by the Central Kalimantan Statistical Bureau (BPS Central Kalimantan 2016) which were supplemented with the health data (e.g. number of live birth, number of registered patients) from the Central Kalimantan Health Department (Dinas Kesehatan Provinsi Kalimantan Tengah 2016; The Indonesian Ministry of Health 2016). We calculated the number of deaths in each village by multiplying the value of the death rate 0.0058 with the total population of each village. We then defined the number of mortality for each health case by multiplying the total number of deaths in each village with the percentage of deaths for the related health case categories obtained from IHME-GHDx Data 2017 (IHME-GHDx 2018). Specifically for Central Kalimantan, the percent of deaths in 2015 for all ages are 33%, 4% and 2% for the related health case categories of cardiovascular, chronic respiratory and lung cancer, respectively. The performance health case categories, the percent of deaths, the relative risk functions and the age group and its fraction values are described in Table 1. A sensitivity analysis was conducted by changing the relative risk function as linear exposure formula, i.e. RR = exp[β (X − X_o_)] as well as varying *X* (the decreasing and increasing) by 10 μg/m^3^ and *X*_*o*_ at 10 μg/m^3^ (the lowest level according to WHO 2016).

**Table 1:** The potential health case categories, percent of deaths, age groups and fraction values, and relative risk function to PM_2.5_

Health case categories	Percent of deaths from exposure to PM_2.5_ for all ages (%)^a^	RR function and *β* coefficient for PM_2.5_^b^	Age group and fraction (%)
Premature mortality (all-cause)^c^	100	Linear exposure; 0.001829	All ages (100%)
Chronic respiratory^c^	4	Linear exposure; 0.003794	Children < 5 years (10%)
Cardiovascular	33	Log-linear exposure; 0.15515	Adults 30 and above (44.5%)
Lung cancer	2	Log-linear exposure; 0.23218	Adults 30 and above (44.5%)

^a^The percent of deaths in 2015 for all ages in Central Kalimantan based on IHME-GHDx (2018)

^b^The suggested *β* coefficients are based on Ostro (2004)

^c^We renormalize the suggested *β* coefficient of PM_10_ by multiplying with the Indonesia conversion factor 48/21 (PM_10_/PM_2.5_ ratio) (WHO 2014)

## Results

### Smoke dispersion and associated PM_2.5_ concentrations from the peat smoke

Based on our spatial analysis, the average of annual mean concentration of PM_2.5_ from the smokes due to peatland fires in Central Kalimantan in the period 2011–2015 was 26 μg/m^3^ (ranging from 4 to 103 μg/m^3^ on the village-based analysis). This is more than twice the recommended WHO AQG annual mean for PM_2.5_ concentration exposure limit which is 10 μg/m^3^ (WHO 2006). Among all regencies in Central Kalimantan, Palangka Raya City showed the highest annual mean of PM2.5 concentrations with the average level of 38 μg/m^3^ (ranging from 27 to 43 μg/m^3^ on the village-based analysis) over the 5-year period. Notably, the average concentration of PM_2.5_ in Central Kalimantan for the year 2015 alone (the year with the highest peatland fire occurrence) was 48 μg/m^3^ (ranging from 40 to 190 μg/m^3^), while in Palangka Raya alone was 65 μg/m^3^ (ranging from 53 to 84 μg/m^3^). It is noted that the study by Koplitz et al. (2016) estimated the average of PM_2.5_ concentration across Indonesia, Malaysia and Singapore due to the 2015 fires was ~ 60 μg/m^3^ (over the 2 month period September–October 2015).

Our analysis also shows that fires in both deep and shallow peatlands in Central Kalimantan are important sources of air pollution (see Appendix 1). Fires from deep and shallow peat contribute roughly the same to the annual mean increase in PM_2.5_ concentration, i.e. both contribute approximately 13 μg/m^3^ (ranging from 2 to 131 μg/m^3^ for the deep peatlands, and ranging from 0.7 to 50 μg/m^3^ for the shallow peatlands). During the 5-year period 2011–2015 from peatland fires, 99% of total villages (1554 of 1569 villages) showed an average annual mean PM_2.5_ concentration above 10 μg/m^3^. Among the 1569 villages in Central Kalimantan, 4 villages (all are in Kapuas regency) showed annual mean PM_2.5_ concentrations above 80 μg/m^3^ (ranging from 82 to 103 μg/m^3^ on the village-based analysis). This means the 4 villages (with the total population of 5886 inhabitants) experience annual mean PM_2.5_ concentrations that exceed more than eight times the exposure limit of PM_2.5_ concentration indicated by the WHO AQGs. We have noted that some villages (including the 4 villages with the annual mean PM_2.5_ concentrations above 80 μg/m^3^) are also located in ex-Mega Rice Project’s area with intensive peatland utilisations over the last decade. In Central Kalimantan during 2011–2015, the total number of hotspots that occurred in deep peatlands was 8% greater than that occurred in shallow peatlands—hence, both shallow and deep peatlands contribute substantially to the health effects resulting from peat fires.

**Fig. 2:**
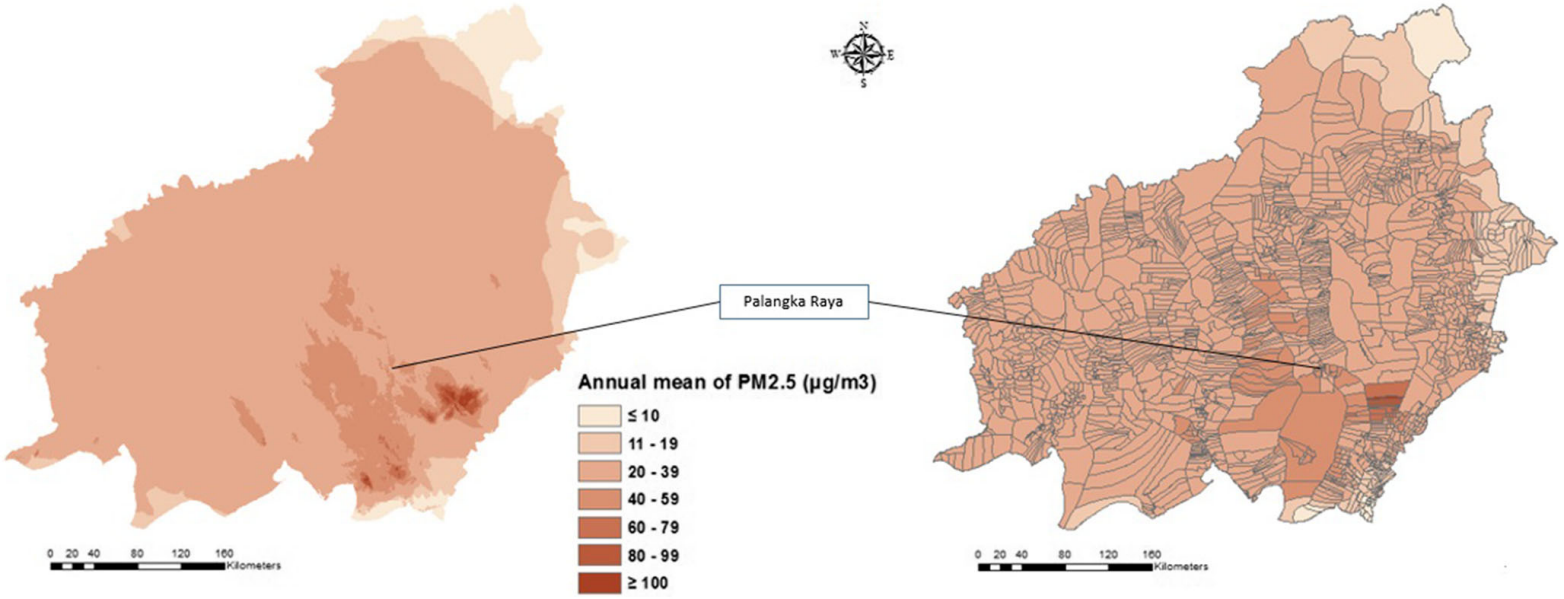
Smoke dispersion and associated average increase in annual mean PM_2.5_ concentrations (μg/m^3^) in Central Kalimantan, Indonesia, from hotspots in peatlands during a 5-year period (2011–2015); the right-hand map is based on 1569 village boundaries

Figure 2 displays the distribution of the annual mean value of PM_2.5_ concentration based on village boundaries in Central Kalimantan from one aggregated plumes of hotspots located in the peatland areas. Our spatial analysis revealed that only 1% of total villages (15 of 1569 villages) in Central Kalimantan showed low PM_2.5_ concentrations (less than 10 μg/m^3^) in accordance with the exposure limit for PM_2.5_ suggested by WHO AQG. More concerning, about 78% of total villages in Central Kalimantan (1230 of 1569) experience PM_2.5_ exposures with annual average PM_2.5_ concentrations ≥ 20 μg/m^3^. This means that about 2 million inhabitants (about 80% of the total population in Central Kalimantan), including more than 430 thousand children aged 5 to 14 years, 65 thousand infants aged between 0 and 1 year, and over 1.4 million adult people aged 27 years and older, experience health impacts due to the inhalation of PM_2.5_.

### Potential human health outcomes

Table 2 summarizes the potential health outcomes with the number of premature mortality and disease cases experienced by the local populations in Central Kalimantan due to long-term exposure to PM_2.5_ with an increase in annual mean PM_2.5_ concentration of 26 μg/m^3^ during 2011 to 2015. Appendix 2 presents the sensitivity analysis for changing the relative risk function and varying *X* and *X*_*o*_ (concentrations of PM_2.5_).

**Table 2:** The potential health outcomes due to exposure to PM_2.5_ emissions from peat smoke in Central Kalimantan, Indonesia, during a 5-year period (2011–2015)

Health case categories	Relevant age group and number of population in age group (people)	Estimated number of deaths for all ages	Estimated number of deaths due to peat smoke	Estimated number of deaths due to peat smoke per 100,000 people in age group
Premature mortality (all-cause) - of which, due to:	All ages (2.5 × 10^6^)	14,601	648	26
Chronic respiratory	All ages (2.5 × 10^6^)	584	55	2
	Children < 5 years (2.5 × 10^5^)	58	6	2
Cardiovascular	All ages (2.5 × 10^6^)	4818	266	11
	Adults 30 and above (1.1 × 10^6^)	2144	119	11
Lung cancer	All ages (2.5 × 10^6^)	292	95	4
	Adults 30 and above (1.1 × 10^6^)	130	42	4

We estimate that the long-term exposure to PM_2.5_ from peat smoke, as estimated during a 5-year period (2011–2015), causes 648 premature mortality cases per year (26 mortality cases per 100,000 people). These include 55 mortality cases due to chronic respiratory diseases, 266 mortality cases from cardiovascular diseases and 95 mortality cases from lung cancer. It is noted that the mortality cases due to chronic respiratory diseases include 6 mortality cases of children aged below 5 years (this equates to 2 mortality cases per 100,000 children aged below 5 years).

Our sensitivity analysis revealed that with an increase of 10 μg/m^3^ in the PM_2.5_ concentration, the premature mortality cases increase with 34%, while the mortality cases due to chronic respiratory disease, cardiovascular diseases and lung cancer will increase with 27%, 108% and 15%, respectively. With a decrease of 10 μg/m^3^ in the PM_2.5_ concentration, the premature mortality will decrease with 45%, and the mortality due to chronic respiratory disease, cardiovascular diseases and lung cancer will decrease by 47%, 27% and 26%, respectively. Also, the value of RR for premature mortality is ranging from 1.00 to 1.09 within the different exposure functions and background concentrations. The highest numbers of cases were in Kotawaringin Timur regency which has a relatively large population and a high exposure to smog from peat fires.

## Discussion

This present study has several limitations. Uncertainties are generated from the meteorological inventory datasets and the referenced values used as inputs in the HYSPLIT model which are used in the analysis of the average concentration of PM_2.5_ in Central Kalimantan. We recognise that the differences in default inputs among meteorological inventory data (e.g. GDAS 1°, Reanalysis (NCEP/NCAR), GFAS, have different spatial resolutions) cause uncertainty related to the estimation of plume trajectory which then affects the estimation of both PM_2.5_ concentration and its spatial distribution (Khairullah et al. 2017; Koplitz et al. 2016; Crippa et al. 2016). In the HYSPLIT model, the plume trajectories and dispersions of PM_2.5_ are simulated based on the Bluesky model in which the GDAS archive is set up as default input meteorological data. The GDAS data have resulted in the enhanced data assimilation methods, having the highest horizontal, vertical and temporal resolution (Godłowska et al. 2015). Also, using a 1-km^2^ resolution for input values might contribute to the differences in the calculated results of PM_2.5_ concentrations. However, a sensitivity experiment that we conducted by changing a 1-km^2^ resolution to a 2 × 2 km resolution using the same procedure as for the 1-km^2^ model resulted in no significant changes in calculated PM_2.5_ concentrations. The wind directions and topography are the main factors that influence the smoke dispersion and associated distribution of PM_2.5_ concentrations (Khairullah et al. 2017).

Besides, in this study we did not address the smoke dispersion from the neighbouring provinces. Peat fires in adjacent provinces will also contribute to smog in Central Kalimantan. We did not consider this effect, and we are therefore underestimating the health effects from peat fires.

In relation to the health impact estimation, several uncertainties were associated with our assumptions. First, we averaged the smoke concentrations over the year to assess the health effects. However, in reality smoke has a seasonal occurrence. Most of the (thick) smoke occurred from July to November (months when the land clearing activities usually start). We are not able to indicate if taking an annual average is leading to an over- or underestimate of the mortality and morbidity assessment. Our study also assessed the average of the annual mean of PM_2.5_ concentration in 2011–2015 which included an El Niño period. The El Niño period (e.g. 2015) has months with an extreme reduction of precipitation and heavy fire activity and risks. Nevertheless, fires in peatland areas have occurred during non-drought years as well (see Gaveau et al. 2014). Our assumption is that the period 2011 to 2015 is representative. We cannot be sure that this is the case. However, we note that land conversion of peatlands in Central Kalimantan is still ongoing which implies that future smog may be worse than present conditions.

Second, we used the logarithm exposure function by Ostro (2004) to estimate the health impacts. The logarithm functions are recommended by WHO to estimate the health impacts in the areas with the high concentrations of air pollution (Burnett et al. 2014). However, the uncertainty on the estimation will be related to the unknown parameters such as the suggested *β* coefficients for PM_2.5_ in this model. These parameters were estimated from the American Cancer Society (ACS) cohort studies (Ostro 2004; WHO 2006). This uncertainty can lead to different outcomes when the coefficients are not consistent with the risk model form (Burnett et al. 2014; Héroux et al. 2015). Thus, conducting proper epidemiological studies in the area is recommended in order to refine the exposure functions especially for the purpose of evaluating the impacts of episodic severe smoke from landscape fires. It is noted that we were not able to assess the morbidity impacts (such as cardiovascular diseases, lung diseases and lost working days) since the baseline data for the occurrence of such diseases is missing in Kalimantan.

Third, we calibrated our model based on air quality data available for only one city, i.e. Palangka Raya. No other data points are available in Central Kalimantan, in spite of the significant health risks related to peat fires, as indicated by our study. We therefore recommend the Government of Indonesia to expand the number of air quality monitoring stations in the province.

In order to assess the accuracy of our study, we compared our findings with available reported data on health impacts. The Central Kalimantan government reported (without mentioned specific data per case) that 2483 people (including 407 infants) died in 2015 (Dinas Kesehatan Provinsi Kalimantan Tengah 2016). However, causes for mortality were not specified. There were also no reports on the occurrence of air pollution–related diseases, even though newspaper reports in 2015 reported increases in hospital admissions (Dinas Kesehatan Provinsi Kalimantan Tengah 2016). It is noted that the Indonesia Government reported the total number of mortality cases in the whole of Indonesia due to the 2015 haze caused by forest and land fires to be 19 people, with more than 500,000 cases of acute respiratory infections (World Bank 2016). In the same report, for Central Kalimantan, the health impacts of forest and peatland fires in 2015 were reported to be only 1 mortality case and nearly 25,000 cases of upper respiratory tract infections (BNPB Indonesia 2017; The Indonesian Ministry of Health 2015). However, our study shows that this is an underestimate of the actual health impacts of fires, which is related to the government only analysing the short-term health effects of exposure to fire in a specific year. Also, the latter studies by Koplitz et al. (2016) and Crippa et al. (2016) estimated the health impacts in Indonesia by analysing a short-term period of the 2015 haze event caused by forest and land fires (September–October 2015). Koplitz et al. estimated 91,600 excess mortality for the Indonesian population aged over 25 years with the average PM_2.5_ concentrations of ~60 μg/m^3^, while Crippa et al. estimated 11,880 excess all-cause premature mortalities due to short-term exposure to unhealthy air quality conditions (using simulated 24-hr PM_2.5_ of 56–160 μg/m^3^) and ~ 75,600 excess all-cause premature mortalities due to long-term exposure to the PM_2.5_ concentrations for the overall population in Indonesia, Malaysia and Singapore (including 3223 premature mortality cases due to lung cancer in the adult population aged over 25 years). In our study, we calculate the long-term health effects of the recurrent annual exposure to smoke from the peat fires (based on average fire and smoke conditions over 2011-2015). We cannot scale up our results to the whole of Indonesia given that the smoke concentration varies considerably over the different islands, but note that Central Kalimantan has a relatively low population of only 2.5 million, only 1% of the country’s population.

A range of studies show that the long-term exposure to PM_2.5_ is a main driver for the health effects of air pollution (e.g. see Burnett et al. 2018; Hoek et al. 2013; Pope et al. 2002; Pope and Dockery 1999). Even though the fires of 2015 were large compared with these of preceding years, also in other years the people of Central Kalimantan are exposed to smoke from peat fires. This study shows the importance of considering these long-term health effects.

## Conclusions

Our study estimated the long-term health impacts of frequent exposure to high PM_2.5_ concentration on the human population in Central Kalimantan due to smoke and peatland fires. We model fire and smoke occurrence in the period 2011–2015 and assume that this period is representative for people’s long-term exposure. We showed that the 2.5 million people in Central Kalimantan are exposed to annual mean PM_2.5_ concentrations, due to peat fires, that are well above the WHO AQG of 10 μg/m^3^. The average increase in annual mean PM_2.5_ concentrations due to peat fires (in shallow and deep peat) in Central Kalimantan was 26 μg/m^3^, of which the annual mean PM_2.5_ concentrations from hotspots in deep peat were 13 μg/m^3^ (ranging from 2 to 131 μg/m^3^) and from the shallow peat were also 13 μg/m^3^ (ranging from 0.7 to 50 μg/m^3^). This long-term exposure of PM_2.5_ from recurrent peat fires and smoke events causes 648 premature mortality cases per year which includes 55 mortality cases due to chronic respiratory diseases, 266 mortality cases due to cardiovascular diseases and 95 mortality cases due to lung cancer. This equates to 26 premature mortality cases per 100,000 people.

The assessment of long-term health impacts on the local population may help the local government and stakeholders in Central Kalimantan province to better assess the health implications of different peatland uses and to take the initiatives to set and enforce higher standards for sustainable peatland management (particularly mitigation policies on fires and drained peatland uses; and also adding air quality monitoring stations). Although the results of our study cannot be extrapolated, it still indicates that a large number of fatalities due to peat fires may occur in Indonesia at large. There are about 57 million inhabitants in Sumatra and about 16 million inhabitants in Kalimantan, and most of these are affected on an annual basis by smoke from burning peatlands. Our work confirms the high urgency of addressing the ongoing peatland conversion and degradation in Indonesia.

## Figures and Tables

**Fig. 3:**
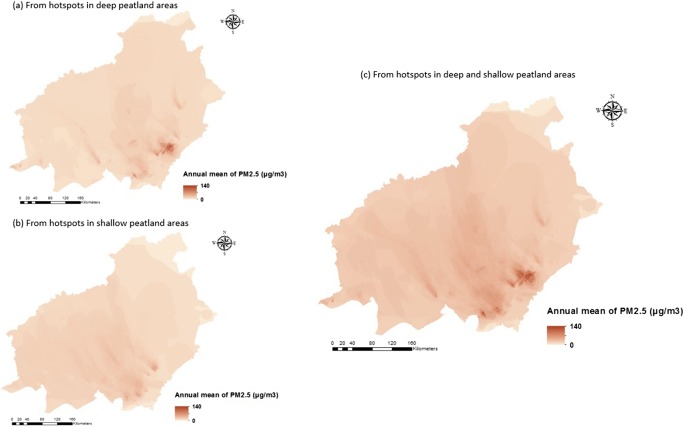
Smoke dispersion and associated annual average concentration of PM_2.5_ (μg/m^3^) in Central Kalimantan, Indonesia, from hotspots 2011–2015 in (a) deep peatland areas; (b) shallow peatland areas; (c) deep and shallow peatland areas combined

## Appendix 2

**Table 3:** The health impacts due to exposure to PM_2.5_ emissions from peat smoke in Central Kalimantan, Indonesia, during a 5-year period (2011–2015) (sensitivity analysis, showing impacts of 10 μg/m^3^ increase and decrease in PM_2.5_ concentrations, as well as double the PM_2.5_ concentration)

Health case categories	*X* ^a^ (μg/m^3^ PM_2.5_)	*X*_*o*_^a^ (μg/m^3^ PM_2.5_)	Shape of exposure function, RR and attributable fraction for PM_2.5_	Number of deaths from exposure to PM_2.5_ for all ages
Premature mortality (all-cause)	26 26 26 26 36 16 52 26 26 26 26 36 16 52	4 5.8 8 10 4 4 4 4 5.8 8 10 4 4 4	Linear; 1.04; 0.04 Linear; 1.04; 0.04 Linear; 1.03; 0.03 Linear; 1.03; 0.03 Linear; 1.06; 0.06 Linear; 1.02; 0.02 Linear; 1.09; 0.09 Log-linear; 1.00; 0.003 Log-linear; 1.00; 0.002 Log-linear; 1.00; 0.002 Log-linear; 1.00; 0.002 Log-linear; 1.00; 0.004 Log-linear; 1.00; 0.002 Log-linear; 1.00; 0.004	648 598 514 463 871 359 1303 46 37 30 25 54 32 63
Chronic respiratory	26 26 26 26 36 16 52 26 26 26 26 36 16 52	4 5.8 8 10 4 4 4 4 5.8 8 10 4 4 4	Linear; 1.09; 0.09 Linear; 1.08; 0.08 Linear; 1.07; 0.07 Linear; 1.06; 0.06 Linear; 1.13; 0.12 Linear; 1.05; 0.05 Linear; 1.21; 0.17 Log-linear; 1.01; 0.01 Log-linear; 1.01; 0.01 Log-linear; 1.00; 0.004 Log-linear; 1.00; 0.003 Log-linear; 1.01; 0.01 Log-linear; 1.00; 0.004 Log-linear; 1.01; 0.01	55 51 42 38 70 29 102 4 3 2 2 4 3 5
Cardiovascular^b^	26 26 26 26 36 16 52 26 26 26 26 36 16 52	4 5.8 8 10 4 4 4 4 5.8 8 10 4 4 4	Linear; 1.23; 0.23 Linear; 1.21; 0.21 Linear; 1.18; 0.15 Linear; 1.16; 0.13 Linear; 1.34; 0.25 Linear; 1.12; 0.1 Linear; 1.57; 0.35 Log-linear; 1.3; 0.05 Log-linear; 1.24; 0.08 Log-linear; 1.18; 0.15 Log-linear; 1.15; 0.13 Log-linear; 1.36; 0.11 Log-linear; 1.2; 0.04 Log-linear; 1.44; 0.19	1151 1056 766 693 1242 543 1741 266 386 765 637 553 194 930
Lung cancer^b^	26 26 26 26 36 16 52 26 26 26 26 36 16 52	4 5.8 8 10 4 4 4 4 5.8 8 10 4 4 4	Linear; 1.34; 0.34 Linear; 1.31; 0.31 Linear; 1.27; 0.2 Linear; 1.24; 0.18 Linear; 1.52; 0.33 Linear; 1.18; 0.14 Linear; 1.91; 0.45 Log-linear; 1.47; 0.32 Log-linear; 1.37; 0.27 Log-linear; 1.29; 0.22 Log-linear; 1.23; 0.18 Log-linear; 1.59; 0.37 Log-linear; 1.31; 0.23 Log-linear; 1.72; 0.42	104 95 63 57 100 45 137 95 81 66 57 109 70 124

^a^*X* is the observed concentration of PM_2.5_ (in μg/m^3^) and *X*_*o*_ is the background concentration (μg/m^3^, as the lowest effect level)

^b^The suggested *β* coefficients for measuring cardiovascular case and lung cancer case in the linear exposure function approach are 0.00893 and 0.01267, respectively, based on Ostro (2004)

## References

[CR1] Atwood EC, Englhart S, Lorenz E, Halle W, Wiedemann W, Siegert F (2016). Detection and characterization of low temperature peat fires during the 2015 fire catastrophe in Indonesia using a new high-sensitivity fire monitoring satellite sensor (FireBird). PLoS ONE.

[CR2] Benjamin PYHL, Zoe GD, Matthew JS (2017). Smoke pollution disrupted biodiversity during the 2015 El Niño fires in Southeast Asia. Environ Res Lett.

[CR3] BNPB Indonesia (Badan Nasional Penanggulangan Bencana Indonesia) (2017) Data Informasi Bencana Indonesia: Kebakaran Hutan dan Lahan di Provinsi Kalimantan Tengah Tahun 2015. Available online: http://bnpb.cloud/dibi/tabel1b (Accessed on 19 September 2018)

[CR4] BPS Central Kalimantan (2016) Provinsi Kalimantan Tengah Dalam Angka Kalimantan Tengah Province in Figures 2016. BPS Statistics (Badan Pusat Statistik) of Kalimantan Tengah Province. Palangka Raya Available online: https://kalteng.bps.go.id/publication/2016/07/15/8c86b2a27099d5760635d2b0/provinsi-kalimantan-tengah-dalam-angka-2016.html (Accessed on 11 July 2017)

[CR5] BPS Central Kalimantan (2017) CDR crude death rate 2015 in Parameter Hasil Proyeksi Penduduk Kalimantan Tengah 2010-2035*.* BPS Statistics (Badan Pusat Statistik) of Kalimantan Tengah Province. Palangka Raya. Available online: https://kalteng.bps.go.id/statictable/2017/04/27/409/parameter-hasil-proyeksi-penduduk-kalimantan-tengah-tahun-2010-2035.html (Accessed on 31 May 2019)

[CR6] Burnett RT, Pope CA, Ezzati M, Olives C, Lim SS, Mehta S, Anderson HR (2014). An integrated risk function for estimating the global burden of disease attributable to ambient fine particulate matter exposure. Environ Health Perspect.

[CR7] Burnett R, Chen H, Szyszkowicz M, Fann N, Hubbell B, Pope CA, Apte JS, Brauer M, Cohen A, Weichenthal S, Coggins J, Di Q, Brunekreef B, Frostad J, Lim SS, Kan H, Walker KD, Thurston GD, Hayes RB, Lim CC, Turner MC, Jerrett M, Krewski D, Gapstur SM, Diver WR, Ostro B, Goldberg D, Crouse DL, Martin RV, Peters P, Pinault L, Tjepkema M, Donkelaar A, Villeneuve PJ, Miller AB, Yin P, Zhou M, Wang L, Janssen NAH, Marra M, Atkinson RW, Tsang H, Thach TQ, Cannon JB, Allen RT, Hart JE, Laden F, Cesaroni G, Forastiere F, Weinmayr G, Jaensch A, Nagel G, Concin H, Spadaro JV (2018). Global estimates of mortality associated with long-term exposure to outdoor fine particulate matter. Proc Natl Acad Sci.

[CR8] Carmenta R, Zabala A, Daeli W, Phelps J (2017). Perceptions across scales of governance and the Indonesian peatland fires. Glob Environ Chang.

[CR9] Crippa P, Castruccio S, Archer-Nicholls S, Lebron GB, Kuwata M, Thota A, Sumin S, Butt E, Wiedinmyer C, Spracklen DV (2016). Population exposure to hazardous air quality due to the 2015 fires in Equatorial Asia. Sci Rep.

[CR10] Dinas Kesehatan Provinsi Kalimantan Tengah (2016) Profil Kesehatan 2015 Provinsi Kalimantan Tengah. Available online: http://www.depkes.go.id/resources/download/profil/PROFIL_KES_PROVINSI_2015/21_KALTENG _2015.pdf (Accessed on 11 Sept 2018)

[CR11] Gaveau DLA, Salim MA, Hergoualc'h K, Locatelli B, Sloan S, Wooster M, Marlier ME, Molidena E, Yaen H, DeFries R, Verchot L, Murdiyarso D, Nasi R, Holmgren P, Sheil D (2014). Major atmospheric emissions from peat fires in Southeast Asia during non-drought years: evidence from the 2013 Sumatran fires. Sci Rep.

[CR12] Giglio L. MODIS Collection 6 Active Fire Product User’s Guide Version A (2015) Department of Geographical Sciences. University of Maryland. Available online: http://modis-fire.umd.edu/pages/manuals.php (Accessed on 25 July 2016)

[CR13] Godłowska J, Hajto MJ, Tomaszewska AM (2015). Spatial analysis of air masses backward trajectories in order to identify distant sources of fine particulate matter emission. Arch Environ Prot.

[CR14] Héroux ME, Anderson HR, Atkinson R, Brunekreef B, Cohen A, Forastiere F, Hurley F, Katsouyanni K, Krzyzanowski DK, Künzli N, Mills I, Querol X, Ostro B, Walton H (2015). Quantifying the health impacts of ambient air pollutants: recommendations of a WHO/Europe project. Int J Public Health.

[CR15] Hirano T, Segah H, Kusin K, Limin S, Takahashi H, Osaki M (2012). Effects of disturbances on the carbon balance of tropical peat swamp forests. Glob Chang Biol.

[CR16] Hoek G, Krishnan RM, Beelen R, Peters A, Ostro B, Brunekreef B, Kaufman JD (2013). Long-term air pollution exposure and cardio- respiratory mortality: a review. Environ Health.

[CR17] Huijnen V, Wooster MJ, Kaiser JW, Gaveau DLA, Flemming J, Parrington M, Inness A, Murdiyarso D, Main B, van Weele M (2016). Fire carbon emissions over maritime southeast Asia in 2015 largest since 1997. Sci Rep.

[CR18] IHME-GHDx (2018) Institute for Health Metrics and Evaluation (IHME) - Global Health Data Exchange (GHDx): Global Burden of Disease Study 2017 (GBD 2017) Results. Global Burden of Disease Collaborative Network. Seattle, United States. Available from http://ghdx.healthdata.org/gbd-results-tool (Accessed on 20 May 2019)

[CR19] INCAS (2016) Indonesian National Carbon Accounting System. Available online: http://www.incas-indonesia.org/data/central-kalimantan/ (Accessed on 14 Feb 2017).

[CR20] Khairullah, Effendy S, Makmur EES (2017). Trajectory and concentration PM 10 on forest and vegetation peat-fire HYSPLIT model outputs and observations (period: September – October 2015). IOP Conf Ser Earth Environ Sci.

[CR21] Koplitz SN, Mickley LJ, Marlier ME, Buonocore JJ, Kim PS, Liu T, Sulprizio MP, DeFries RS, Jacob DJ, Pongsiri M, Myers SS, Schwartz J (2016) Public health impacts of the severe haze in Equatorial Asia in September–October 2015: Demonstration of a new framework for informing fire management strategies to reduce downwind smoke exposure. Environ Res Lett 11(9). 10.1088/1748-9326/11/9/094023

[CR22] Lohberger S, Stängel M, Atwood EC, Siegert F (2018). Spatial evaluation of Indonesia’s 2015 fire-affected area and estimated carbon emissions using Sentinel-1. Glob Chang Biol.

[CR23] Marlier ME, DeFries RS, Kim PS, Koplitz SN, Jacob DJ, Mickley LJ, Myers SS (2015). Fire emissions and regional air quality impacts from fires in oil palm, timber, and logging concessions in Indonesia. Environ Res Lett.

[CR24] Miettinen J, Shi C, Liew SC (2016). Land cover distribution in the peatlands of Peninsular Malaysia, Sumatra and Borneo in 2015 with changes since 1990. Glob Ecol Conserv.

[CR25] Miettinen J, Hooijer A, Vernimmen R, Liew SC, Page SE (2017). From carbon sink to carbon source: Extensive peat oxidation in insular Southeast Asia since 1990. Environ Res Lett.

[CR26] MoEFRI (Ministry of Environment and Forestry Republic of Indonesia) (2015) Sipongi: Karhutla Monitoring Sistem. Ministry of Environment and Forestry Republic of Indonesia. Jakarta, Indonesia. Available online: http://sipongi.menlhk.go.id/peta/hotspot_provinsi (Accessed on 26 Oct 2016)

[CR27] MoFRI (Ministry of Forestry Republic of Indonesia) (2014) Land cover maps. Ministry of Forestry Republic of Indonesia. Jakarta, Indonesia. Available online: http://webgis.menlhk.go.id:8080/pl/pl.htm (Accessed on 14 Feb 2015)

[CR28] Ostro B (2004) Outdoor air pollution: assessing the environmental burden of disease at national and local levels. Environmental Burden of Disease Series, No. 5 (Prüss-Üstün A, Campbell-Lendrum, D, Corvalán C, Woodward A, eds). Geneva: World Health Organization. Available: https://www.who.int/quantifying_ehimpacts/publications/ebd5.pdf (accessed 31 May 2019).

[CR29] Pope CAIII, Dockery DW, Holgate ST, Samet JM, Koren HS, Maynard RL (1999). Epidemiology of particle effects. Air pollution and health.

[CR30] Pope CA, Burnett RT, Thun MJ, Calle EE, Krewski D, Ito K, Thurston GD (2002). Lung cancer, cardiopulmonary mortality, and long-term exposure to fine particulate air pollution. JAMA.

[CR31] Ritung S, Wahyunto, Nugroho K, Sukarman, Hikmatullah, Suparto CT (2011) Peta lahan gambut Indonesia. Skala 1:250.000 (Maps of Peatland Distribution in Indonesia). Balai Besar Sumber Daya Lahan Pertanian (BBSDLP) Ministry of Agriculture, Republic of Indonesia

[CR32] Ruchi S, Rajasekhar B (2017). Indoor human exposure to size-fractionated aerosols during the 2015 Southeast Asian smoke haze and assessment of exposure mitigation strategies. Environ Res Lett.

[CR33] Stein AF, Draxler RR, Rolph GD, Stunder BJB, Cohen MD, Ngan F (2015). NOAA’s HYSPLIT atmospheric transport and dispersion modelling system. Bull Am Meteorol Soc.

[CR34] Stockwell CE, Jayarathne T, Cochrane MA, Ryan KC, Putra EI, Saharjo BH, Nurhayati AD, Albar I, Blake DR, Simpson IJ, Stone EA, Yokelson RJ (2016). Field measurements of trace gases and aerosols emitted by peat fires in Central Kalimantan, Indonesia during the 2015 El Niño. Atmos Chem Phys.

[CR35] Sumarga E (2017). Spatial indicators for human activities may explain the 2015 fire hotspot distribution in Central Kalimantan Indonesia. Ecol Soc.

[CR36] Tacconi L (2016). Preventing fires and haze in Southeast Asia. Nat Clim Chang.

[CR37] The Indonesian Ministry of Health (2015) Health problems caused by smoke haze from the 2015 forest and peat fires. Available online: http://www.depkes.go.id/resources/download/pusdatin/infodatin/infodatin-asap.pdfhttp://www.depkes.go.id/resources/download/pusdatin/infodatin/http://www.pusdatin.kemkes.go.id/ folder/view/01/structure-publikasi-pusdatin-info-datin.html (Accessed on 8 Aug 2016)

[CR38] The Indonesian Ministry of Health (2016) Indonesia health profile 2015: Profil Kesehatan Indonesia 2015. Available online: http://www.depkes.go.id/resources/download/pusdatin/profil-kesehatan-indonesia/profil-kesehatan-Indonesia-2015.pdf (Accessed on 8 Aug 2017)

[CR39] The Indonesian National Institute of Aeronautics and Space (2015) The estimate of the Indonesian burnt areas in the period 1 July – 20 October 2015. Available online: https://www.lapan.go.id/index.php/subblog/read/ 2015/2052/LAPANPerkirakan-Luas-dan-Sebaran-Daerah-Terbakar-di-Indonesia. http://pusfatja.lapan.go.id/ files_uploads_ebook/publikasi/Panduan_hotspot_2016% 20versi%20draft%201_LAPAN.pdf. (Accessed on 8 Aug 2016)

[CR40] Turetsky MR, Benscoter B, Page S, Rein G, van der Werf GR, Watts A (2015). Global vulnerability of peatlands to fire and carbon loss. Nat Geosci.

[CR41] Uda SK, Hein L, Sumarga E (2017). Towards sustainable management of Indonesian tropical peatlands. Wetl Ecol Manag.

[CR42] Uda SK, Schouten G, Hein L (2018) The institutional fit of peatland governance in Indonesia. Land Use Policy Elsevier Ltd. 10.1016/j.landusepol.2018.03.031

[CR43] WHO Regional Office for Europe (2016) Health risk assessment of air pollution – general principles. Copenhagen. Available online http://www.euro.who.int/__data/assets/pdf_file/0006/298482/Health-risk-assessment-air-pollution-General-principles-en.pdf?ua=1 (Accessed on 27 Mar 2019).

[CR44] WHO World Health Organization (2006). Air quality guidelines for particulate matter, ozone, nitrogen dioxide and sulfur dioxide - Global update 2005 - Summary of risk assessment. Environ Sci Pollut Res.

[CR45] WHO World Health Organization (2013) Health effects of particulate matter: policy implications for countries in eastern Europe, Caucasus and central Asia. Available online: http://www.euro.who.int/__data/assets/pdf_file/ 0006/189051/Health-effects-of-particulate-matter-final-Eng.pdf (Accessed on 14 Feb 2017)

[CR46] WHO World Health Organization (2014) Ambient Air Pollution Database 2014. Available online: http://www. who.int/quantifying_ehimpacts/national/.../AAP_PM_database_May2014.xls (Accessed on 27 January 2018)

[CR47] World Bank (2016) The cost of fire : an economic analysis of Indonesia’s 2015 fire crisis. Indonesia sustainable landscapes knowledge. Note no. 1. Washington, D.C. World Bank Group. Available online: http://pubdocs.worldbank.org/en/643781465442350600/Indonesia-forest-fire-notes.pdf. (Accessed on 8 Aug 2017)

